# *gmmDenoise*: A New Method and *R* Package for High‐Confidence Sequence Variant Filtering in Environmental DNA Amplicon Analysis

**DOI:** 10.1111/1755-0998.70023

**Published:** 2025-08-04

**Authors:** Yusuke Koseki, Hirohiko Takeshima, Ryuji Yoneda, Kaito Katayanagi, Gen Ito, Hiroki Yamanaka

**Affiliations:** ^1^ Department of Life Design, Faculty of Home Economics Otsuma Women's University Chiyoda‐ku Tokyo Japan; ^2^ Department of Marine Biology, School of Marine Science and Technology Tokai University Shimizu‐ku Shizuoka Japan; ^3^ Faculty of Marine Science and Technology Fukui Prefectural University Obama Fukui Japan; ^4^ Fukui Prefectural Satoyama‐Satoumi Research Institute Wakasa Fukui Japan; ^5^ Faculty of Advanced Science and Technology Ryukoku University Otsu Shiga Japan; ^6^ Center for Biodiversity Science Ryukoku University Otsu Shiga Japan

**Keywords:** amplicon sequence variants, eDNA metabarcoding, haplotyping, intraspecific diversity, population genetics, sequence filtering

## Abstract

Assessing and monitoring genetic diversity is vital for understanding the ecology and evolution of natural populations but is often challenging in animal and plant species due to technically and physically demanding tissue sampling. Although environmental DNA (eDNA) metabarcoding is a promising alternative to the traditional population genetic monitoring based on biological samples, its practical application remains challenging due to spurious sequences present in the amplicon data, even after data processing with the existing sequence filtering and denoising (error correction) methods. Here we developed a novel amplicon filtering approach that can effectively eliminate such spurious amplicon sequence variants (ASVs) in eDNA metabarcoding data. A simple simulation of eDNA metabarcoding processes was performed to understand the patterns of read count (abundance) distributions of true ASVs and their polymerase chain reaction (PCR)‐generated artefacts (i.e., false‐positive ASVs). Based on the simulation results, the approach was developed to estimate the abundance distributions of true and false‐positive ASVs using Gaussian mixture models and to determine a statistically based threshold between them. The developed approach was implemented as an *R* package, *gmmDenoise* and evaluated using single‐species metabarcoding datasets in which all or some true ASVs (i.e., haplotypes) were known. Example analyses using community (multi‐species) metabarcoding datasets were also performed to demonstrate how *gmmDenoise* can be used to derive reliable intraspecific diversity estimates and population genetic inferences from noisy amplicon sequencing data. The *gmmDenoise* package is freely available in the GitHub repository (https://github.com/YSKoseki/gmmDenoise).

## 要旨

遺伝的多様性の評価とモニタリングは、自然集団の生態や進化を理解する上で不可欠であるが、動植物種においては技術的および物理的に組織試料採取の労力が大きいため、しばしば困難である。環境DNA(eDNA)メタバーコーディングは、生物試料に基づく従来の集団遺伝学的モニタリングに代わる有望な手法であるが、既存の配列フィルタリングやノイズ除去(エラー補正)手法を用いたデータ処理を行った後でも、アンプリコンデータ内に偽の配列が含まれるため、実用化には依然として課題がある。本研究では、eDNAメタバーコーディングデータにおいて、このような偽のアンプリコン配列変異(ASVs)を効果的に除去できる新規のアンプリコンフィルタリング手法を開発した。まず、eDNAメタバーコーディングプロセスの簡易シミュレーションを実施し、真のASVsとポリメラーゼ連鎖反応(PCR)によって生成されたアーティファクト(すなわち偽陽性ASVs)のリード数(頻度)分布のパターンを解析した。このシミュレーション結果に基づき、ガウス混合モデルを用いて真のASVsと偽陽性ASVsの頻度分布を推定し、それらの間における統計ベースの閾値を決定するアプローチを開発した。開発した手法は、*R*パッケージ ***gmmDenoise*** として実装し、すべてまたは一部の真正ASVs(すなわちハプロタイプ)が判明している一種系メタバーコーディングデータセットを用いて評価を行った。さらに、群集(多種系)メタバーコーディングデータセットを用いた事例解析を行い、*gmmDenoise*によりノイズの多いアンプリコン配列データから信頼できる種内多様性の推定値や集団遺伝学的推論を引き出す方法を示した。 ***gmmDenoise*** パッケージは、GitHubリポジトリ(https://github.com/YSKoseki/gmmDenoise)で自由に利用可能である。

## 1 Introduction

The magnitude, structure and dynamics of genetic diversity, a primary source of information about biological populations, can provide insights into the evolutionary history of populations (e.g., natural selection and genetic drift), their present status (size and connectivity) and future prospects (viability) (Frankham et al. 2010; Cutter 2019; Kardos et al. 2021). These insights are crucial for advancing the ecological and evolutionary understanding of populations and devising effective population management and conservation strategies (for case studies, see Frankham et al. 2010, 2017; Allendorf et al. 2022). However, obtaining genetic diversity data from wild animal and plant populations can be challenging, as sampling individual organisms and their tissues can be technically and physically demanding and therefore logistically costly, particularly in elusive animal species with cryptic behaviour and mobile life history (e.g., Sigsgaard et al. 2016; Parsons et al. 2018). In addition, collecting numerous biological samples may be legally and ethically unacceptable in endangered and threatened species (e.g., Urban et al. 2023; Wakimura et al. 2023).

Environmental DNA (eDNA) technologies have revolutionised biodiversity research by enabling the simultaneous detection of multiple species (or, strictly speaking, genetically distinct taxonomic groups) and the noninvasive characterisation of biological communities. In particular, the development of eDNA metabarcoding, or polymerase chain reaction (PCR) amplification of DNA fragments present in environmental samples (e.g., water or sediments) followed by high‐throughput sequencing of amplicons, has facilitated this approach (Coissac et al. 2012; Deiner et al. 2017; Ruppert et al. 2019). As eDNA metabarcoding has become widely used in the biodiversity monitoring of species and higher‐order taxonomic groups, research has begun to explore its applicability to intraspecific genetic diversity monitoring (Adams et al. 2019; Sigsgaard et al. 2020). For example, Sigsgaard et al.'s (2016) pioneering study showed that metabarcoding of seawater samples targeting polymorphic regions of species‐specific mitochondrial DNA (mtDNA) of the whale shark ( *Rhincodon typus* ) recovered haplotype frequencies similar to those obtained from tissue samples. Subsequent studies have also shown that eDNA metabarcoding can be used to assess genetic diversity within a species or a closely related group of species (e.g., emperor fish, *Lethrinus* spp., Stat et al. 2017; the harbour porpoise, *Phocoena phocoena* , Parsons et al. 2018; the ayu fish, *Plecoglossus altivelis* , Tsuji, Maruyama, et al. 2020; Tsuji, Miya, et al. 2020; Pacific salmon and trout, *Oncorhynchus* spp., Weitemier et al. 2021; the blackfoot pāua, *Haliotis iris* , Adams et al. 2023). Furthermore, a few attempts have been made to harness the power of eDNA metabarcoding to simultaneously infer intraspecific genetic diversity in multiple species (stream macroinvertebrates, Elbrecht et al. 2018; Zizka et al. 2020; marine benthic communities, Turon et al. 2020).

Although previous studies have shown promise for the development of eDNA metabarcoding‐based genetic diversity monitoring, the practical application of this approach remains challenging. One of the major challenges is the occurrence of spurious sequences. Most of such artefact sequences are point errors caused by PCR and sequencing, whereas others are PCR‐mediated recombinants, or chimaeras (Edgar and Flyvbjerg 2015; Tikhonov et al. 2015). These sequences may have a relatively minor effect on the characterisation of species diversity, as most of them differ from the correct sequences by only a single or a few nucleotides (Edgar and Flyvbjerg 2015; Tikhonov et al. 2015) and are likely to be correctly assigned to a species or taxonomic group. In the assessment of intraspecific genetic diversity, however, these artefacts will be regarded as genetic variants (i.e., haplotypes) and can seriously affect the genetic variation data and their analyses (Cummings et al. 2010). Current approaches to dealing with artefact amplicon sequence variants (ASVs), or false‐positive haplotypes, include sequence filtering with commonly used but arbitrary authenticity criteria and thresholds (e.g., removal of ASVs occurring in only one sample, represented by < 10 reads, or representing < 1% of total reads per sample) and model‐based sequence error correction, often referred to as denoising (e.g., *DADA2*, Callahan et al. 2016; *UNOISE3*, Edgar 2016; *DnoisE*, Antich et al. 2022) (for additional approaches, see Parsons et al. 2018; Bennington et al. 2024). Despite these approaches being used in combination with each other as the best strategy, the processed data often contain a considerable proportion of false‐positive haplotypes (e.g., Elbrecht et al. 2018; Tsuji, Miya, et al. 2020; Macé et al. 2022), indicating the need to develop alternative or complementary approaches.

In this study, we developed a novel sequence filtering approach that can effectively remove spurious sequences from eDNA metabarcoding data using Gaussian mixture models (GMMs; McLachlan and Peel 2000). We first present a simulation analysis of eDNA metabarcoding processes to determine the characteristics of read count (abundance) distributions of true haplotypes and corresponding artefacts (i.e., false‐positive haplotypes) in the amplicon data. We then introduce our GMM‐based amplicon filtering approach that was devised on the basis of the simulation results and its *R* package implementation, *gmmDenoise* (https://github.com/YSKoseki/gmmDenoise). We briefly describe the functions of gmmDenoise, which include GMM estimation of abundance distributions of true and false‐positive haplotypes, statistical determination of the abundance filtering threshold, and their visualisation. We further evaluate the performance of *gmmDenoise* using single‐species metabarcoding datasets in which all or some true haplotypes are known. Finally, we present example analyses of community metabarcoding datasets to show how *gmmDenoise* can be used to derive accurate intraspecific diversity estimates and population genetic inferences from noisy amplicon sequencing data.

## 2 Materials and Methods

### 2.1 Simulation Analysis

All the existing ASV filtering and denoising approaches described above implicitly or explicitly use the notion that spurious sequences have a lower abundance than true sequences. For our new approach, we developed a more quantitative version of this notion by performing a simulation of eDNA metabarcoding processes using models with literature‐based parameter values. While eDNA metabarcoding consists of several processes—DNA extraction from environmental samples, PCR amplification of the extracts, sequencing of PCR amplicons and bioinformatics analysis of amplicon sequences—our simulation focused primarily on PCR because it is likely to be a significant source of artefacts, as multiple cycles (typically, 30–35) of amplification can repeatedly introduce errors into the amplified sequences and multiplicatively increase the number of artefact sequences (Eckert and Kunkel 1991).

First, we generated a simulated sample of an eDNA extract. We assumed that the extract contained 10,000 reads from 100 unique sequence variants with a 160‐bp length. The nucleotide sequences in the variants were arbitrary, with the four bases randomly assigned under the constraint of 56% GC content. Following Kelly et al.'s (2019) simulation study, which used proportional rather than absolute biomass of species to flexibly capture changes in community structure in competitive PCR, we modelled the sequence variants *i* as having differing proportions of initial abundance $a_{i0}$ at cycle *j* = 0 defined by a symmetric Dirichlet distribution,

(1) $$a_{i0}∼\text{Dirichlet}\left(\gamma \right)$$

with *γ* = 1. By definition, $\sum_{i=1}^{100}a_{i0}=1$.

We next subjected the simulated eDNA sample to PCR amplification with cycle number *j* = 1–35, and modelled each cycle as follows:

(2) $$a_{\mathrm{ij}}^{\prime }=\frac{a_{\mathrm{ij}-1}p_{i}\varepsilon }{\sum_{i=1}^{I}a_{\mathrm{ij}-1}}$$

(3) $$a_{\mathrm{ij}}=a_{\mathrm{ij}-1}+a_{\mathrm{ij}}^{\prime }$$

where $a_{\mathrm{ij}}^{\prime }$ is the proportional abundance of newly generated amplicon reads of a sequence *i*, for *i* = 1, …, *I*, after cycle *j*; $a_{\mathrm{ij}-1}$ is the total amplicon abundance of the sequence after the previous cycle; $p_{i}$ is the amplification efficiency for the sequence; and *ε* is a small stochastic error. Note that the number of unique sequences *I* is 100 at *j* = 0 but will increase in the later cycles due to the occurrence of artefact sequences as described below. We modelled the sequence‐specific amplification efficiency by a beta distribution:

(4) $$p_{i}\sim \text{Beta}\left(\alpha ,\beta \right)$$

with *α* = *β* = 5, which gives a mean of 0.5 and a standard deviation of 0.15. For *ε*, we used a lognormal distribution, $\varepsilon \sim \text{Lognormal}\left(\mu ,\sigma^{2}\right)$ with *μ* = 0 and *σ* = 0.05. Although proportional abundance is convenient for capturing changes in amplicon composition, it is unsuitable for simulating the discrete event of the occurrence of an artefact sequence. Therefore, we calculated the absolute abundance corresponding to $a_{\mathrm{ij}}^{\prime }$ and $a_{\mathrm{ij}}$, $A_{\mathrm{ij}}^{\prime }$ and $A_{\mathrm{ij}}$ as $A_{\mathrm{ij}}^{\prime }=\text{floor}\left(A_{\mathrm{ij}-1}a_{\mathrm{ij}}^{\prime }\right)$ and $A_{\mathrm{ij}}=A_{\mathrm{ij}-1}+A_{\mathrm{ij}}^{\prime }$, respectively, with $A_{i0}=\text{floor}\left(10,000a_{i0}\right)$.

We simulated the occurrence of artefact sequences during PCR as follows: PCR can generate various errors including substitutions, insertions, deletions and chimaeras. However, given their highest occurrence rate (Potapov and Ong 2017), we assumed for simplicity that only substitution errors could occur in our simulation. This assumption would result in fewer unique artefact sequences than would be the case if all possible errors were taken into account; nevertheless, the simulation result would represent the general pattern of read count distribution of artefact sequences. Again, for simplicity we considered the number of substitution errors as the number of artefact sequences, assuming only one substitution in an amplified sequence during a PCR cycle. This assumption seems to be reasonable because the substitution rates per base per doubling event reported for high‐fidelity polymerases (Potapov and Ong 2017) are sufficiently low (10^−7^ to 10^−5^) for the sequence length of 160 bp. With these assumptions, we modelled the proportional abundance of artefact sequences derived from template sequence *i* at cycle *j* as follows:

(5) $$e_{\mathrm{ij}}^{\prime }=La_{\mathrm{ij}}^{\prime }\xi$$

where *L* is the sequence length (160 bp) and *ξ* is a substitution error rate defined by a beta distribution, $\xi \sim \text{Beta}\left(36,3\times 10^{6}\right)$, which has a mean ($1.2\times 10^{-5}$) and a standard deviation ($0.2\times 10^{-5}$) corresponding to the estimates reported for a commonly used high‐fidelity polymerase, KOD (Potapov and Ong 2017). Similar to the absolute number of amplicon reads, the absolute number of artefact sequences derived from template *i* is given as $E_{\mathrm{ij}}^{\prime }=\text{floor}\left(A_{\mathrm{ij}-1}e_{\mathrm{ij}}^{\prime }\right)$. The summation of this value across all templates $\sum_{i=1}^{I}E_{\mathrm{ij}}^{\prime }$ is the total number of artefact sequences newly generated during a PCR cycle. In each artefact sequence, a substitution error was introduced at a random position *s* drawn from a discrete uniform distribution, $s\sim \text{dunif}\left(1,160\right)$. Because most base substitutions are transitions, or interchanges between purines (A, G) or pyrimidines (T, C), with transversions occurring less frequently (Potapov and Ong 2017), we considered only transitions to occur in the simulated substitutions. The variants of artefact sequences derived from a common template *k*, for *k* = 1, …, $E_{\mathrm{ij}}^{\prime }$, share the proportional abundance $e_{\mathrm{ij}}^{\prime }$, so that the abundance of each variant $e_{\mathrm{ijk}}^{\prime }$ is given as $e_{\mathrm{ijk}}^{\prime }=e_{\mathrm{ij}}^{\prime }/E_{\mathrm{ij}}^{\prime }$. The amplification efficiencies of the artefact sequence variants were assumed to be the same as that of the original template sequence $p_{i}$ because of their high sequence identity. At the end of each PCR cycle, we dereplicated the amplified sequences and merged their abundances, considering the possible occurrence of identical mutations and reverse mutations.

After the PCR simulation, we simulated DNA sequencing of PCR amplicons. It can be modelled as a multinomial sampling of *N* sequencing reads for *I* sequences (i.e., 100 true sequences and artefacts derived from them) according to the sequences' proportional abundance ***a*** (bolding indicates vectors), so that:

(6) $$R\sim \text{Multinomial}\left(\frac{a}{\sum_{i=1}^{I}a_{i}},N\right)$$

where ***R*** is a vector of the observed read count for *I* sequences. We used $N=1.5\times 10^{6}$, assuming an average Illumina MiSeq sequencing run.

We performed 100 iterations of the eDNA metabarcoding simulation described above and used them to analyse the read count distributions of true and artefact sequences. To visually characterise the distributions, we used histograms and kernel density estimates. The simulation and analysis were performed in *R* ver. 4.1.2 (R Core Team 2021) using packages *tidyverse* ver. 1.3.1 (Wickham et al. 2019), *doParallel* ver. 1.0.14 (Microsoft Corporation and Weston 2018) and *dirmult* ver. 0.1.3.5 (Tvedebrink 2010). The scripts used are available at the GitHub repository, https://github.com/YSKoseki/gmmDenoise_Paper and Zenodo deposit (Koseki et al. 2025).

### 2.2 *gmmDenoise* : Package Description and Performance Evaluation

The characteristic patterns of read count distribution of artefact sequences in the simulation (see Section 3) gave us an idea of a new amplicon filtering approach: to use GMMs to estimate the read count (abundance) distributions of true and false‐positive ASVs so that a statistically based abundance filtering threshold between the distributions can be determined. To implement this approach, we developed an *R* package *gmmDenoise* (GitHub repository, https://github.com/YSKoseki/gmmDenoise). The package comprises several functions for parameter estimation and visualisation of ASV abundance distributions. The main function *gmmem* fits *k*‐component GMMs to ASV abundance data using the expectation–maximisation (EM) algorithm implemented in the *normalmixEM* function of the *mixtools* package (Benaglia et al. 2009). Taking a vector of ASV abundance and an integer for the number of mixture components *k* as arguments, *gmmem* returns a list object (class *gmmem*) containing the estimates of means, standard deviations and mixing proportions for the fitted Gaussian mixture components. The returned *gmmem*‐class object is used in the *quantile.gmmem* function to obtain a vector of confidence limits (CLs) for the mixture components (default is upper one‐sided 95% CLs). As shown in the simulation (see Section 3), the parameter‐estimated mixture components represent the abundance distributions of true ASVs and artefacts derived from them, with the uppermost component corresponding to the former and the other component(s) corresponding to the latter. Therefore, the CL value of the second uppermost component can be used as a statistically validated abundance filtering threshold between the true and false‐positive ASVs. To select *k*, which is key in GMM analysis, two different approaches are provided, two‐fold cross‐validation and sequential parametric bootstrap testing, which are implemented in the *gmmcv* and *gmmbs* functions, respectively. To facilitate graphical representations of the above analyses, *gmmDenoise* provides ready‐to‐use plotting functions that extend the *autoplot* function in the *ggplot2* package (Wickham 2016): *autoplot.gmmcv*, *autoplot.gmmbs* and *autoplot.gmmem*. An example workflow of analysis using these parameter estimation and visualisation functions is presented in the package repository referenced above. The same repository also includes a description of best practices, which outlines our recommended workflow for applying gmmDenoise in combination with other tools to analyse datasets that span multiple sequencing runs. As noted in the best practice description, we recommend applying gmmDenoise separately to each sequencing run. While a more stringent approach might involve applying it to individual samples within a run, our preliminary analyses (data not shown) suggested that this was not practical—most likely due to insufficient information on the ASV read count distribution within single samples.

To evaluate the performance of *gmmDenoise*, we used two eDNA metabarcoding datasets of *P. altivelis* published by Tsuji, Miya, et al. (2020) and Tsuji, Maruyama, et al. (2020), which are publicly available at the NCBI Sequence Read Archive (SRA) with BioProject accession numbers PRJDB6813 and PRJDB8911, respectively. Both datasets (referred to as single‐species datasets 1 and 2, hereafter) consist of amplicon sequences obtained by *P. altivelis‐*specific PCR amplification followed by Illumina MiSeq sequencing, targeting a 166‐bp fragment of the mtDNA control region. They, however, differ in that dataset 1 is from a mock sample comprising only known haplotypes (a mixture of rearing water samples) (Tsuji, Miya, et al. 2020), whereas dataset 2 is from multiple field (stream water) samples for which a non‐exhaustive list of reference haplotypes obtained from fish samples is available (Tsuji, Maruyama, et al. 2020). The original authors used these datasets to show that, although the major denoising algorithms *DADA2* and *UNOISE3* considerably decreased the number of false‐positive ASVs, the accuracy of *DADA2*‐ or *UNOISE3*‐denoised data can be further improved by additional filtering using the detection rate of each ASV in technical (i.e., PCR) or field sample (glass fibre filter) replicates as the filtering criterion. Tsuji, Miya, et al. (2020) proposed discarding ASVs with a < 100% detection rate in technical replicates (i.e., using only ASVs that are found in all replicates), which we considered to be a good comparison for our *gmmDenoise* method.

We applied similar analytical procedures to both single‐species datasets 1 and 2 to obtain *P. altivelis* ASVs. Primer trimming of raw sequence reads (FASTQ files) was performed using *Cutadapt* ver. 3.5 (Martin 2011). The primer‐trimmed reads were processed by one of three bioinformatics pipelines with or without the use of *DADA2* and *UNOISE3*. In both denoising methods, the same values of stringency‐controlling parameters as in the original studies (Tsuji, Miya, et al. 2020; Tsuji, Maruyama, et al. 2020) were used: in *DADA2*, the default values (OMEGA_A = 1e‐40 and OMEGA_C = 1e‐40) were used for both datasets, whereas in *UNOISE3 α* values were 2 for dataset 1 and 3 for dataset 2. The *DADA2* denoising was performed with the *DADA2* package ver. 1.22.0 (Callahan et al. 2016), and the *UNOISE3* denoising was performed using the *R* implementation function of the *UNOISE3* algorithm, *Denoise* in the *JAMP* package ver. 0.77 (Elbrecht et al. 2018). For read processing without the use of denoising methods, Tsuji, Miya, et al. (2020) and Tsuji, Maruyama, et al. (2020) used their custom pipelines; instead, we used *JAMP*'s *Denoise* function with an extremely low‐stringency setting of *α* (10,000), so the function was virtually ineffective. The amplicon reads obtained by each bioinformatics pipeline were dereplicated into ASVs, which were taxonomically classified in *Claident* ver. 0.9.2022.01.26 (Tanabe and Toju 2013) to confirm their identity as *P. altivelis* . The ASV abundance data and taxonomy table generated were integrated into a *phyloseq* (McMurdie and Holmes 2013) object in *R* using the *speedyseq* package ver. 0.5.3.9018 (McLaren 2022).

The three *P. altivelis* ASV datasets obtained (i.e., non‐denoised, *DADA2*‐denoised and *UNOISE3*‐denoised) were then analysed and filtered using the *gmmDenoise* package ver. 0.3.1. These procedures included the following steps: the construction of a histogram of ASV abundance distribution, selection of the optimum *k* value for the ASV abundance distribution based on visual inspection of the histogram and cross‐validation analysis, GMM estimation of the true and false‐positive ASV abundance distributions with the selected *k*, determination of the CL‐based ASV abundance threshold and removal of ASVs with an abundance value lower than the determined threshold. The accuracy of the three ASV datasets filtered by *gmmDenoise* was compared with that of the corresponding datasets filtered by Tsuji, Miya, et al.'s (2020) detection rate‐based method described above. All scripts used are available at the GitHub repository and Zenodo deposit referenced above.

### 2.3 Examples

To provide illustrative examples of how our *gmmDenoise* filtering method can derive accurate intraspecific diversity estimates and population genetic inferences from community eDNA datasets, we analysed the datasets reported by Nakagawa et al. (2018) and Ahn et al. (2020) and available at SRA with BioProject accession numbers PRJDB5158 and PRJDB10433, respectively. Both datasets are among the recent outcomes of eDNA‐based fish community monitoring using the fish universal primer set MiFish‐U/E targeting a 163–185 bp fragment of the mitochondrial 12S ribosomal RNA gene (Miya et al. 2020), but they differ in the target ecosystems (streams and estuaries) and therefore in fish species.

We analysed the two datasets (referred to as stream and estuarine fish community datasets, hereafter) using a procedure similar to that for the single‐species dataset analyses: primer trimming; read processing and merging with three pipelines (*DADA2* denoising, *UNOISE3* denoising and no denoising); taxonomic assignment; integration of sequence abundance data and taxonomy table; and *gmmDenoise*‐based sequence filtering. However, *UNOISE3* denoising was performed with *α* = 5 (default for the *JAMP*'s *Denoise* function; Elbrecht et al. 2018) instead of 2 because our preliminary analysis revealed that denoising with the latter value was too stringent, leading to only a small number of ASVs retained and consequently to a less informative read count distribution for the subsequent *gmmDenoise* analysis (Figure S1). Read processing without denoising was also performed differently from the single‐species analyses: the publicly available MiFish primer–based eDNA metabarcoding pipeline (Sato et al. 2018) was used according to the original studies (Nakagawa et al. 2018; Ahn et al. 2020). In each of the three ASV datasets, we calculated the total numbers of fish species and fish ASVs detected before and after *gmmDenoise* filtering to assess the filtering effect.

After the above whole‐community analysis, we used the *DADA2*‐denoised dataset to perform intraspecific population genetic analysis on the Japanese torrent catfish ( *Liobagrus reinii* ) in the stream fish community dataset and flathead grey mullet ( *Mugil cephalus* ) in the estuarine fish community dataset because phylogenetic and phylogeographic patterns were available for these two example species (Nakagawa et al. 2016; Shen et al. 2011), providing references for our analysis. Using all fish ASVs obtained without the use of *gmmDenoise* filtering and the geographically relevant reference sequences of the example species from GenBank, we constructed a maximum‐likelihood (ML) phylogenetic tree (under the GTR + I + G nucleotide‐substitution model) from which the minimum tree (subtree) containing all ASVs of the example species was extracted. The ASV labels on the tree tips were coloured in black if these ASVs were retained by *gmmDenoise* and in red if they were not. To further visualise the results of *gmmDenoise* filtering, we generated an ASV‐by‐sample abundance heatmap based on the log read count, log_10_(read count +1). Finally, we constructed a parsimony haplotype network and haplotype frequency map using only the ASVs retained by *gmmDenoise* to identify the population genetic structure. Tree construction and visualisation were performed using the *R* packages *DECIPHER* ver. 2.22.0 (Wright 2015), *phangorn* ver. 2.8.1 (Schliep 2011) and *ggtree* ver. 3.2.1 (Yu et al. 2017). The abundance heatmap was generated using the *ggplot2* package ver. 3.3.5 (Wickham 2016), and the haplotype network was constructed using the *pegas* package ver. 1.1 (Paradis 2010). The haplotype frequency map was constructed using the packages *sf* ver. 1.0.6 (Pebesma 2018), *rmapshaper* ver. 0.4.6 (Teucher and Russell 2022) and *ggspatial* ver. 1.1.7 (Dunnington et al. 2022) and shape file data from the Digital National Land Information download services, https://nlftp.mlit.go.jp/ksj/. All scripts are available at the above GitHub repository and Zenodo deposit.

## 3 Results

### 3.1 Simulation

**FIGURE 1:**
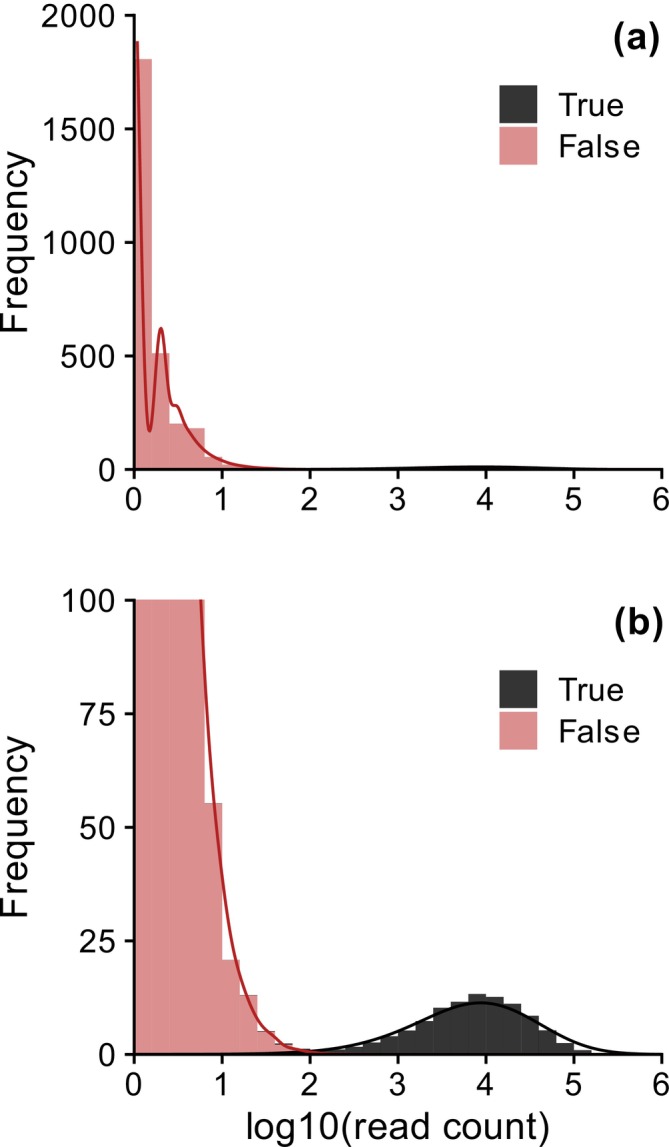
Histograms (bars) and scaled kernel density estimates (lines) representing frequency distributions, that is the distributions of mean read counts for 100 true unique sequences (true haplotypes, shown in black) and PCR‐generated artefacts derived from them (false‐positive haplotypes, shown in red) across 100 simulated PCR replicates: (a) the entire plot and (b) the *y*‐axis zoomed in to highlight the trough between the distributions of true and false‐positive haplotypes.

The simulation of eDNA metabarcoding processes yielded (mean ± SD) 2798 ± 145 unique error sequences (range: 2260–3075) along with the 100 true sequences. Most of the erroneous sequences had a few reads (mean ± SD: 2.0 ± 3.6), but some showed 1–2 orders of magnitude larger read counts (range: 1–429) because they were generated in the earliest PCR cycles and were repeatedly amplified in the later cycles. Consequently, the read count distribution of erroneous sequences was characterised by a primary peak in the lower range with secondary peaks or shoulders in the upper range (Figure 1a). In contrast, true sequences had higher read counts (mean ± SD: 1.5 × 10^4^ ± 2.2 × 10^4^; range: 1–3.5 × 10^4^) and were characterised by a distinct unimodal read count distribution (Figure 1b).

### 3.2 *gmmDenoise* Performance

**FIGURE 2:**
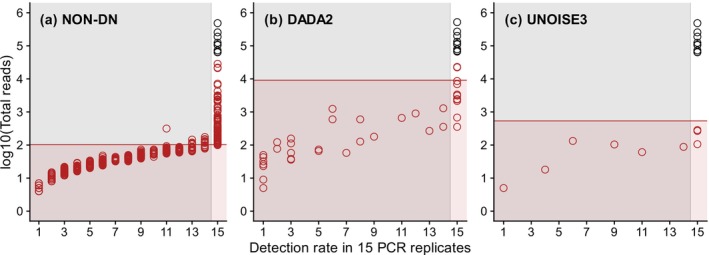
Abundance (log read count)–detection rate plots of amplicon sequence variants (ASVs) representing the effects of *gmmDenoise* filtering versus Tsuji, Miya, et al.'s (2020) detection rate‐based filtering on the single‐species dataset 1 processed with (a) no denoising, (b) *DADA2* denoising or (c) *UNOISE3* denoising. ASVs (haplotypes): black circles, true; red circles, false‐positive. The filtering effects (the areas in which ASVs were inferred as false positives) of *gmmDenoise* and the detection rate‐based method are denoted by red and grey shading, respectively.

The results of our analysis of the single‐species dataset 1 were similar to those of the original study (Tsuji, Miya, et al. 2020) with respect to the number of extracted ASVs, or possible haplotypes. As in Tsuji, Miya, et al. (2020), we recovered all nine true haplotypes from the non‐denoised, *DADA2*‐denoised and *UNOISE3*‐denoised datasets. We identified 1034 false‐positive haplotypes in the non‐denoised dataset, 39 in the *DADA2*‐denoised one, and 9 in the *UNOISE3*‐denoised one; these numbers were comparable to those in the original study (5683, 37 and 10, respectively). As shown by Tsuji, Miya, et al. (2020), the detection rate of each ASV in 15 PCR replicates provided useful information for further filtering of false‐positive haplotypes, but their proposed method of eliminating ASVs that were not detected in at least one PCR replicate did not correctly remove 83 (8.0%) out of 1034, 12 (31%) out of 39, and 3 (33%) out of 9 false positives (Figure 2).

**FIGURE 3:**
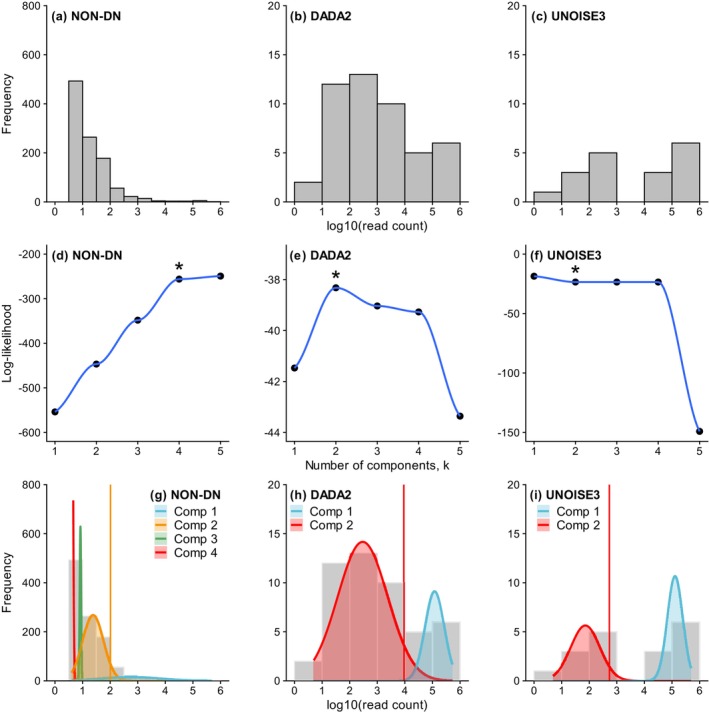
*gmmDenoise* analysis of amplicon sequence variants (ASVs) from the single‐species dataset 1 processed with (a, d, g) no denoising, (b, e, h) *DADA2* denoising and (c, f, i) *UNOISE3* denoising. For each denoising option, visual inspection of read count distribution (a–c) and cross‐validation results (d–f) were used to select the number of mixture components, *k* (marked with an asterisk), with which a Gaussian mixture model was fitted (g–i) to infer a statistically validated cutoff threshold for error filtering, that is the upper one‐sided 95% confidence limit of the second uppermost component (Comp 2), indicated by a vertical line.

In the subsequent *gmmDenoise* analysis, the shapes (i.e., peaks and troughs) of read count distributions (Figure 3a–c) and cross‐validation log‐likelihoods (Figure 3d–f) jointly indicated *k* = 4 for the non‐denoised dataset and *k* = 2 for the *DADA2*‐denoised and *UNOISE3*‐denoised datasets. On the basis of GMMs fitted with these *k* values, the sequence filtering cutoff thresholds were determined as 2.01, 3.96 and 2.73 on the log_10_ scale for the respective datasets (Figure 3g–i). Sequence filtering with the *gmmDenoise*‐determined thresholds had an effect similar to that of the detection rate‐based method in the non‐denoised dataset, as 81 (7.8%) out of 1034 false positives remained after filtering (Figure 2a). In the *DADA2*‐ and *UNOISE3*‐denoised datasets, however, the *gmmDenoise* filtering had a greater effect than the detection rate‐based method, reducing the numbers of false positives from 39 to 2 (5.1%) and from 9 to 0 (0%), respectively (Figure 2b,c). As with the detection rate‐based method, all true haplotypes were correctly retained in all datasets.

**FIGURE 4:**
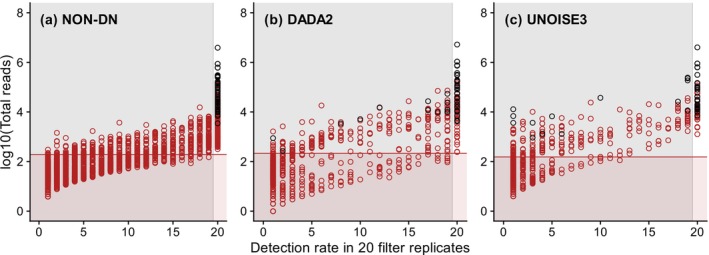
Abundance (log read count)–detection rate plots of amplicon sequence variants (ASVs) representing the effects of *gmmDenoise* filtering versus Tsuji, Miya, et al.'s (2020) detection rate‐based filtering on the single‐species dataset 1 processed with (a) no denoising, (b) *DADA2* denoising or (c) *UNOISE3* denoising. ASVs (haplotypes): black circles, true (known); red circles, possible false‐positives or true unknowns. The filtering effects (the areas in which ASVs were inferred as false positives) of *gmmDenoise* and the detection rate‐based method are denoted by red and grey shading, respectively.

A similar analysis of the single‐species dataset 2 recovered 42, 41 and 38 out of 42 reference haplotypes from the non‐denoised, *DADA2*‐denoised and *UNOISE3*‐denoised datasets, respectively, consistent with the original study (Tsuji, Maruyama, et al. 2020). The corresponding numbers of non‐reference haplotypes (possible false positives) were 4310, 944 and 978, compared to 44,645, 926 and 934 in the original study. The *gmmDenoise* analysis indicated *k* = 2 for the non‐denoised dataset, *k* = 3 for the *DADA2*‐denoised one, and *k* = 2 for the *UNOISE3*‐denoised one (Figure S2). On the basis of GMMs fitted with these *k* values, the log‐scale sequence cutoff thresholds were determined to be 2.28, 2.33 and 2.19 for the respective datasets. Sequence filtering using these thresholds reduced the numbers of non‐reference sequences to 733 (17%), 245 (26%) and 237 (24%) in the respective datasets (Figure 4) without compromising the detection of reference haplotypes, including those with low abundance that were recovered from only one or a few filter replicates after *DADA2* and *UNOISE3* denoising (Figure 4b,c). When the method of eliminating haplotypes that were not detected in at least one filter replicate was used instead, larger proportions of non‐reference haplotypes were removed, leaving only 233 (5%), 57 (6%) and 40 (4%) in the respective datasets (Figure 4). This strong filtering, however, resulted in false negatives, incorrectly removing 12 (30%) out of 41 reference haplotypes in the *DADA2*‐denoised dataset and 14 (37%) out of 38 reference haplotypes in the *UNOISE3*‐denoised datasets (Figure 4b,c).

### 3.3 Examples

Our analysis of the stream fish community dataset using three different pipelines detected a total of 57–60 species per dataset regardless of denoising (Table 1), similar to 55 species detected in the original study (Nakagawa et al. 2018). Similar to the pattern observed in the single‐species analyses, the number of unique ASVs was almost an order of magnitude larger in the non‐denoised dataset than in the two denoised datasets (Table 1; see also Table S1). When sequences were filtered with the *gmmDenoise*‐determined cutoff thresholds (2.44, 2.61 and 2.93 for the non‐denoised, *DADA2*‐denoised and *UNOISE3*‐denoised datasets, respectively; see Figure S3), the numbers of species in the three datasets decreased to 53%–75% and the numbers of ASVs to 15%–37%. The species discarded by *gmmDenoise* included marine species commonly consumed as food, such as *Beryx splendens* , *Cololabis saira* and *Paralichthys olivaceus* (Table S2). In the non‐denoised dataset, a high proportion of ASVs (85%) was removed through *gmmDenoise* filtering, but the number of remaining ASVs was still 74% and 134% higher than those in the *DADA2*‐ and *UNOISE3*‐denoised datasets, respectively.

**TABLE 1:** Total numbers of fish species and amplicon sequence variants (ASVs) detected without and with the use of *gmmDenoise* filtering in three differently processed datasets from stream and estuarine fish community studies.

Study	Without *gmmDenoise*		With *gmmDenoise*	
Dataset	Species	ASV	Species	ASV
Stream fish				
Non‐denoised	60	1198	45 (75%)	176 (15%)
*DADA2*‐denoised	58	275	40 (69%)	101 (37%)
*UNOISE3*‐denoised	57	327	30 (53%)	75 (23%)
Estuarine fish				
Non‐denoised	184	3885	122 (66%)	453 (12%)
*DADA2*‐denoised	182	730	102 (56%)	222 (30%)
*UNOISE3*‐denoised	181	841	100 (55%)	214 (25%)

**FIGURE 5:**
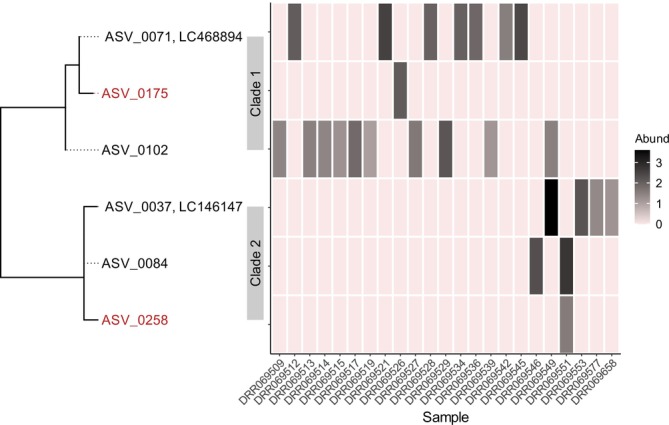
A maximum likelihood phylogenetic tree and abundance (log read count) heatmap of *Liobagrus reinii* amplicon sequence variants (ASVs) extracted from the *DADA2*‐denoised stream fish community dataset. The ASV names shown in red were removed by *gmmDenoise* filtering, and those shown in black were retained.

**FIGURE 6:**
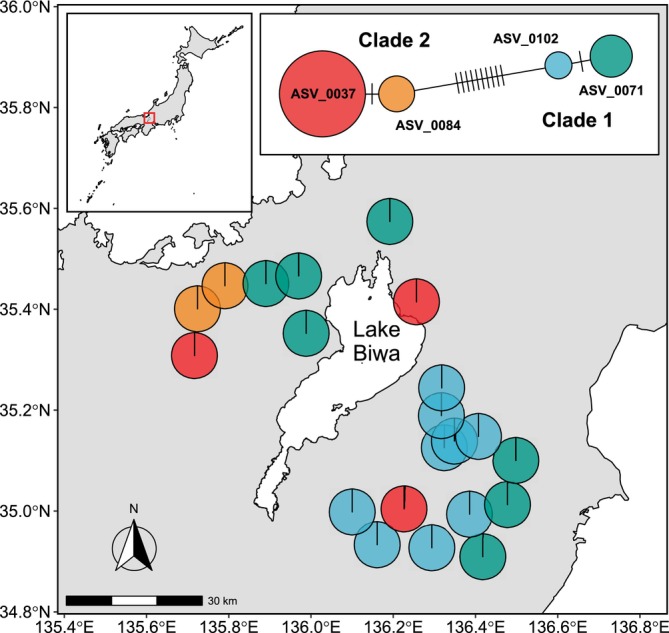
Haplotype map and haplotype network of *Liobagrus reinii* based on amplicon sequence variants (ASVs) extracted from the *DADA2*‐denoised and *gmmDenoise*‐filtered stream fish community dataset. Pie charts on the map represent haplotype frequency (read count) compositions at sites, with different colours used for different haplotypes. In the network, each haplotype is represented by a circle, the colour of which corresponds to the haplotype's colour in the map and the size of which is proportional to the haplotype's total read count. Crosslines on the branches indicate the number of mutational changes between two different haplotypes.

In the population genetic analysis of *L. reinii* using the *DADA2*‐denoised stream fish community dataset, six unique ASVs were extracted without *gmmDenoise* filtering. The *L. reinii* subtree of the ML phylogenetic tree showed that these ASVs and two reference sequences (LC468894 and LC146147) were grouped into two major clades (1 and 2; Figure 5), consistent with the previously observed pattern in the mtDNA cytochrome *b* haplotypes recovered from fish specimens (see figure 2 in Nakagawa et al. 2016). Of the six ASVs, two less abundant ones (ASV0175 and ASV0258), each detected in a single sample, were removed by subsequent *gmmDenoise* filtering (Figure 5). The network of the remaining four *gmmDenoise*‐inferred haplotypes confirmed the two clades identified in the ML tree (Figure 6). Consistent with the known phylogeographic pattern of the distribution of cytochrome *b* gene variants (largely divergent eastern and western Japan clades contact each other in the Lake Biwa region; figure 2 in Nakagawa et al. 2016), our two clades showed overlapping but distinct distributions around Lake Biwa, with clade 1 mostly in the east and clade 2 mostly in the west. Despite the co‐occurrence of four haplotypes from the two clades at the regional scale, almost complete allopatry was identified among haplotypes at the local scale, with most of the sites occupied by only a single haplotype.

Similar to the stream fish community analysis, the estuarine fish community analysis detected a total of 181–184 species regardless of denoising (Table 1), similar to 182 species detected in Ahn et al. (2020). Again, similar to the stream fish community case, the total number of unique ASVs was almost an order of magnitude larger in the non‐denoised dataset than in the two denoised datasets (Table 1; see also Table S3). Sequence filtering with the *gmmDenoise*‐determined cutoff thresholds (2.40, 2.67 and 2.61 for the non‐denoised, *DADA2*‐denoised and *UNOISE3*‐denoised datasets, respectively; see Figure S4) decreased the numbers of detected species to 55%–66% and those of ASVs to 12%–30%. The species discarded by *gmmDenoise* included oceanic species commonly consumed as food, such as *B. splendens* , *Diaphus watasei* and *Oncorhynchus keta* (Table S4). Although *gmmDenoise* filtering discarded a disproportionately large proportion (88%) of ASVs in the non‐denoised dataset, the number of remaining ASVs was still 104% and 112% higher than those in the *DADA2*‐ and *UNOISE3*‐denoised datasets, respectively.

**FIGURE 7:**
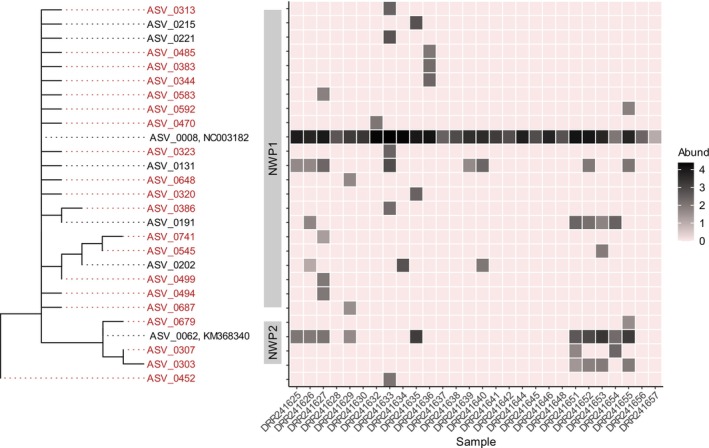
A maximum likelihood phylogenetic tree and abundance (log read count) heatmap of *Mugil cephalus* amplicon sequence variants (ASVs) extracted from the *DADA2*‐denoised estuarine fish community dataset. The ASV names shown in red were removed by *gmmDenoise* filtering, and those shown in black were retained.

**FIGURE 8:**
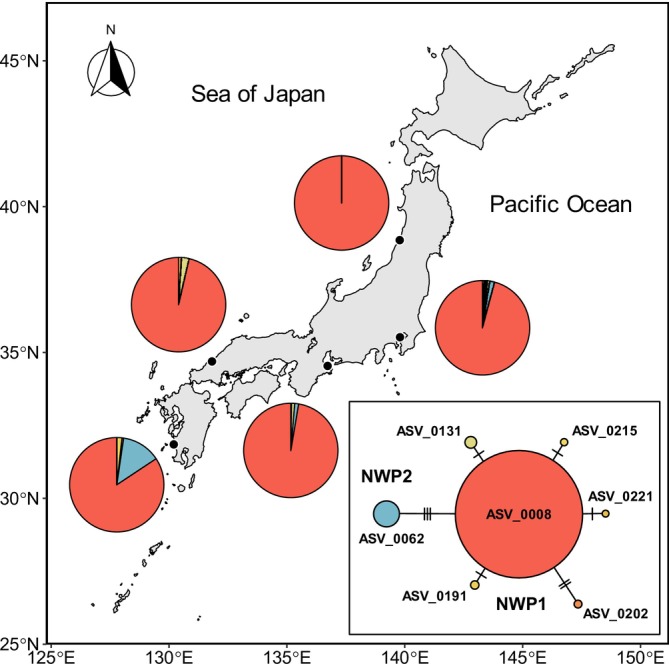
Haplotype map and haplotype network of *Mugil cephalus* based on amplicon sequence variants (ASVs) extracted from the *DADA2*‐denoised and *gmmDenoise*‐filtered estuarine fish community dataset. Pie charts on the map represent haplotype frequency (read count) compositions at sample collection sites (black circles), with different colours used for different haplotypes. In the network, each haplotype is represented by a circle, the colour of which corresponds to the haplotype's colour in the map and the size of which is proportional to the haplotype's total read count. Crosslines on the branches indicate the number of mutational changes between two different haplotypes.

The population genetic analysis of *M. cephalus* identified 27 unique ASVs in the *DADA2*‐denoised dataset without *gmmDenoise* filtering. These ASVs and two reference sequences (NC003182 and KM368340) formed two clades in the ML tree (Figure 7), consistent with the previous analysis of mtDNA cytochrome *c* oxidase I (COI) sequences that showed that the haplotypes from samples collected from Japan and neighbouring regions (Qingdao, China and Primorsky Krai, Russia) were grouped into two clades (NWP1 and NWP2; see figure 2 in Shen et al. 2011). Among the 27 ASVs, *gmmDenoise* filtering removed 20 low‐abundance ASVs. The network of the remaining seven *gmmDenoise*‐inferred haplotypes supported the ML tree as the NWP1 haplotypes formed a cluster of their own, with the main haplotype, ASV0008, connected with the NWP2 haplotype, ASV0062 (Figure 8). ASV0008 occurred at all estuarine sites and dominated the frequency (read count) compositions, while ASV0062 occurred only at the southernmost site and two Pacific coastal sites that are located in the path of the warm Kuroshio Current. These heterogeneous distribution patterns of the NWP1 and NWP2 haplotypes were consistent with the pattern observed in the previous study (see figure 5 in Shen et al. 2011).

## 4 Discussion

We developed a novel GMM‐based approach for metabarcoding ASV filtering to improve the accuracy of eDNA‐based genetic diversity analysis and implemented the corresponding algorithm in the *R* package *gmmDenoise*. The new approach showed high sequence‐filtering performance in the benchmarking analyses of the single‐species datasets, particularly when used in combination with the popular *DADA2* and *UNOISE3* denoising algorithms. Example analyses of the stream and estuarine fish community datasets illustrated the effects of *gmmDenoise* filtering on genetic diversity estimates at the levels of the whole community and individual species. The population genetic patterns of the example species (*L. reinii* and *M. cephalus* ) inferred from the *gmmDenoise*‐filtered sequence data were consistent with those reported in the previous studies. These results show that *gmmDenoise* is a useful option for minimising the confounding effect of errors during PCR amplification and amplicon sequencing and for deriving accurate intraspecific diversity estimates and population genetic inferences from eDNA metabarcoding data.

Our GMM‐based sequence filtering approach is based on a detailed understanding of the read count distribution of erroneous amplicons obtained from a simple simulation of the eDNA metabarcoding processes, which include PCR amplification and amplicon sequencing. This simulation showed that the read counts of erroneous amplicons are, on average, a few orders of magnitude smaller than those of true sequences, providing a quantitative version of the general notion that erroneous sequences have lower abundance than the original true sequences (Edgar and Flyvbjerg 2015; Tikhonov et al. 2015). The simulation results suggest that the read count distribution of erroneous amplicons can be multimodal, with most such amplicons having a small number of reads and some early‐occurring ones having exponentially large read counts. These simulated abundance distribution characteristics of erroneous amplicons provide a solid theoretical basis for our GMM‐based approach that estimates the abundance distributions of true biological sequences and erroneous amplicons derived from them to determine the threshold between them.

Benchmarking analyses using the single‐species datasets 1 and 2 from previous studies (Tsuji, Miya, et al. 2020; Tsuji, Maruyama, et al. 2020) demonstrated the high error‐filtering ability of *gmmDenoise*. Using the first dataset, the original authors (Tsuji, Miya, et al. 2020) showed that their detection rate‐based filtering method (discarding ASVs with < 100% detection rate) significantly reduced the number of false‐positive sequences in the non‐denoised and *DADA2*‐ and *UNOISE3*‐denoised datasets. Using the same dataset, we showed that *gmmDenoise* filtering achieved similar or better performance than the detection rate‐based method, removing a similar number of erroneous sequences in the non‐denoised dataset and a higher number in the denoised datasets. Furthermore, our analysis of the second dataset highlighted that *gmmDenoise* can achieve a balance between the risks of false positives and false negatives. Although *gmmDenoise* filtering removed only a modest number of non‐reference sequences or possible false positives in comparison with the detection rate‐based method, this modest level of filtering had no adverse effect on the recovery of relatively rare (low‐abundance) reference haplotypes, contrasting with the overly strong filtering by the detection rate‐based method, which incorrectly removed one‐third of reference haplotypes in the denoised datasets. As seen in previous studies (Elbrecht et al. 2018; Tsuji, Miya, et al. 2020; Adams et al. 2023), distinguishing true rare haplotypes from false positives is a major bioinformatics challenge because unpredictable sampling bias, PCR error and sequencing error collectively blur the boundary between them. Therefore, the ability of *gmmDenoise* to achieve high error‐filtering performance while maintaining a good balance between false‐positive and false‐negative risks demonstrates its practical usefulness as a sequence filtering method.

In addition to its high performance, *gmmDenoise* offers other advantages. First, it does not require replication‐based information. It is often economically or logistically not feasible to process multiple samples or perform technical replicates, which limits the use of replication‐based approaches, including the detection rate‐based method (Tsuji, Miya, et al. 2020). Relying solely on read count data of unique ASVs, *gmmDenoise* can analyse any amplicon sequencing data and is therefore a practically useful means to improve the accuracy of eDNA metabarcoding data for genetic diversity research and monitoring. Second, a more philosophical advantage of *gmmDenoise* is that it determines filtering thresholds on the basis of statistical principles. As mentioned in the Introduction, abundance filtering has been commonly used to reduce the occurrence of false‐positive sequences, but the abundance threshold is often chosen conventionally or arbitrarily. In contrast, *gmmDenoise* estimates read count distributions of true biological sequences and erroneous amplicons in a GMM framework and uses the upper one‐sided 95% CL of the estimated error distribution as an abundance cutoff threshold (optionally, a more stringent CL can be used). This statistically rational procedure justifies the validity of *gmmDenoise* filtering results and resulting genetic diversity estimates in the downstream analysis. Nevertheless, researchers may wish to adopt a more conservative approach when applying *gmmDenoise*. Such approaches could include testing multiple thresholds (e.g., 95%, 97% and 99% CLs) to assess the robustness of the results and to examine how conclusions may vary depending on the stringency of filtering. Researchers may also compare retained ASVs with known species or haplotype databases, when available, to validate their authenticity and identify potentially spurious sequences.

We consider *gmmDenoise* to be a complementary tool, not an alternative to *DADA2* and *UNOISE*. *gmmDenoise* and the two denoising approaches differ in their strategies: *gmmDenoise* estimates abundance distributions of true and erroneous sequences on the basis of GMMs to determine a threshold between them, whereas *DADA2* and *UNOISE* divide or cluster the unique sequences into groups on the basis of error models that evaluate the likelihood of a unique sequence being an erroneous variant of a similar, more abundant true sequence. Therefore, *gmmDenoise* and the two existing approaches are not methodologically conflicting or redundant. Indicating their complementarity, the single‐species analyses showed that the combined use of *gmmDenoise* filtering and *DADA2* or *UNOISE3* denoising resulted in considerably smaller numbers of false‐positive and non‐reference sequences in the mock and field sample datasets than when the filtering and denoising methods were used separately. Therefore, we suggest that the combined use of *gmmDenoise* and existing denoising methods is the best practice for obtaining accurate amplicon sequence data.

Example analyses of the stream and estuarine fish community datasets illustrate how *gmmDenoise* can be used to derive accurate intraspecific diversity estimates and population genetic inferences from broadly targeted, multispecies eDNA metabarcoding data. In both analyses, *gmmDenoise* filtering removed about one‐third of the total ASVs in the respective *DADA2*‐denoised datasets, including two *L. reinii* ASVs and 20 *M. cephalus* ASVs that were detected in only one or a few samples, respectively. The subsequent population genetic analysis of *L. reinii* based on the four retained ASVs, or *gmmDenoise*‐inferred haplotypes, revealed phylogenetic and phylogeographic patterns consistent with a previous study (Nakagawa et al. 2016), identifying two genetically distant clades co‐occurring regionally with some distribution differences. The analysis also provided a high‐resolution picture of the *L. reinii* population genetic structure, unveiling an almost complete local‐scale segregation of haplotypes. In *M. cephalus* , the analysis based on the seven inferred haplotypes showed low genetic differentiation of the species yet heterogeneous distributions of the haplotypes, consistent with the available phylogenetic and phylogeographic data from a previous study (Shen et al. 2011). These results indicate that *gmmDenoise* holds promise as a tool for accurate population genetic inferences that is widely applicable not only to single‐species amplicon data but also to broadly targeted community metabarcoding data.

Although our approach is promising, its utility and limitations need to be further evaluated with different datasets, particularly those targeting different taxonomic groups other than fishes. One issue that can undermine our approach (and any other PCR‐based approach) is primer bias leading to the consistent over‐ or underrepresentation of a particular group of sequences and therefore biased estimation of the genetic diversity of the population (Kelly et al. 2019). This biased estimation may not have a significant effect on single‐species amplicon data because they are obtained with a species‐specific set of primers, which are likely to amplify all haplotypes within the species with maximal efficiency. However, it can be a major issue in multispecies metabarcoding data, where a broadly targeted universal primer set amplifies sequences from different species with different efficiencies, leading to low read counts and therefore high likelihoods of unidentified haplotypes, or false negatives, in less efficiently amplified species. Thus, despite our promising results in the example fish species, the performance of *gmmDenoise* filtering may vary among different taxonomic groups, depending on the specificity and sensitivity of the available primers. The underestimation of genetic diversity caused by primer bias can be mitigated by using more than one set of primers targeting different gene regions in a multiplex PCR format (Andres et al. 2021; Weitemier et al. 2021). We do not consider bioinformatics approaches to be the only solution to the amplicon sequence filtering issue and other issues in eDNA‐based biodiversity monitoring. Technical aspects of the laboratory analysis of eDNA samples remain to be addressed and can be improved or optimised to obtain higher‐quality amplicon sequence data. Complementary improvements in bioinformatics and laboratory analyses are certainly needed to further enhance the potential of eDNA‐based genetic diversity assessment and population genetic research.

In conclusion, we developed and implemented a novel GMM‐based amplicon sequence filtering approach, *gmmDenoise*, for the accurate eDNA‐based estimation of intraspecific genetic diversity. The *gmmDenoise* method demonstrated high performance with single‐species fish amplicon data and showed great promise in the phylogenetic and phylogeographic analyses of example species from fish community metabarcoding data, especially when combined with the existing denoising algorithms. Although further validations need to be performed in a wide range of taxa, *gmmDenoise* holds potential as a bioinformatics tool for deriving accurate genetic diversity estimates and therefore valid population genetic inferences from increasingly available eDNA metabarcoding data.

## Author Contributions

Yusuke Koseki and Hirohiko Takeshima conceived and designed the study. Yusuke Koseki, Ryuji Yoneda and Kaito Katayanagi performed the analyses. Yusuke Koseki wrote the manuscript. All authors contributed to interpreting the results and improving the manuscript.

## Disclosure

Benefit‐sharing statement: This study used publicly available data that were originally obtained from research conducted in Japan by researchers affiliated with Japanese institutions. As such, the Nagoya Protocol is not applicable. As part of our commitment to benefit‐sharing and advancing eDNA research, we have provided an open‐access *R* package (*gmmDenoise*) that facilitates reliable filtering of sequence variants.

## Conflicts of Interest

The authors declare no conflicts of interest.

## Supporting information


**Data S1:** men70023‐sup‐0001‐DataS1.pdf.

**Data S2:** men70023‐sup‐0002‐DataS2.xlsx.

## Data Availability

All data used for analyses are from the previously published studies and are publicly available at NCBI SRA as referenced in the main text (BioProject accession numbers: PRJDB6813, PRJDB8911, PRJDB5158 and PRJDB10433). All scripts are available at the GitHub repository, https://github.com/YSKoseki/gmmDenoise_Paper, with snapshots archived in the Zenodo repository (https://doi.org/10.5281/zenodo.15016880). The *gmmDenoise* package is available at https://github.com/YSKoseki/gmmDenoise (https://doi.org/10.5281/zenodo.15015857).
