# Supplementary material for: gmmDenoise: A New Method and R Package for High‐Confidence Sequence Variant Filtering in Environmental DNA Amplicon Analysis
Source: Mol Ecol Resour. 2025 Aug 4;25(8):e70023. doi: 10.1111/1755-0998.70023 (PMC12550461; doi:10.1111/1755-0998.70023)
Supplement: Supplementary file 1 — Data S1: men70023‐sup‐0001‐DataS1.pdf. [file MEN-25-e70023-s001.pdf]

MOLECULAR ECOLOGY RESOURCES

Supporting Information for:

gmmDenoise: a new method and *R* package for high-confidence sequence variant filtering in environmental DNA amplicon analysis

Yusuke Koseki, Hirohiko Takeshima, Ryuji Yoneda, Kaito Katayanagi, Gen Ito, Hiroki Yamanaka

Table of Contents:

**FIGURE S1**	Page 2
**FIGURE S2**	Page 3
**FIGURE S3**	Page 4
**FIGURE S4**	Page 5

NB: Supplementary tables are presented in a separate Excel file.

MOLECULAR ECOLOGY RESOURCES

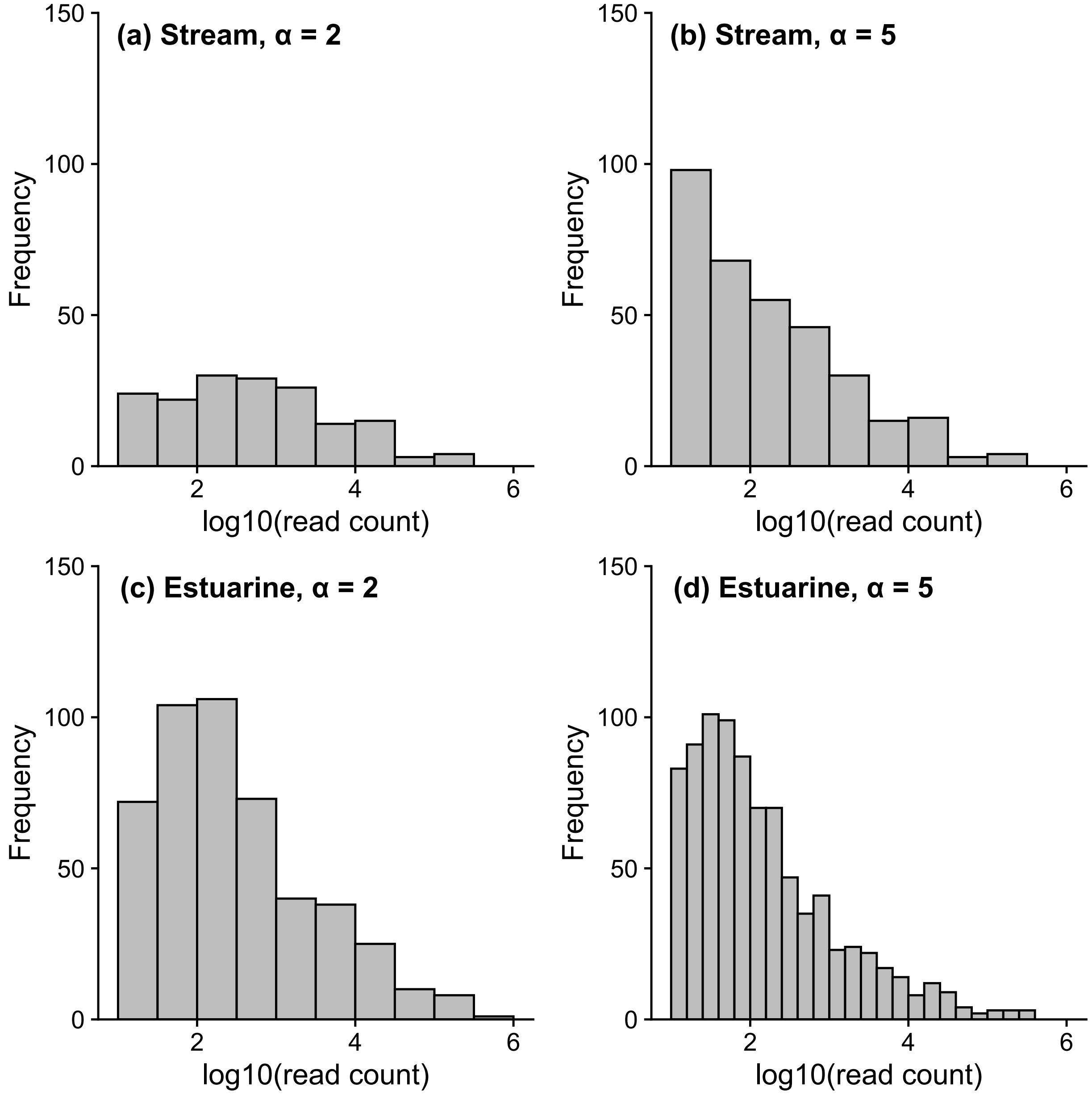

FIGURE S1 Read count distributions of fish amplicon sequence variants (ASVs) extracted from (a, b) the stream fish community dataset and (c, d) the estuarine fish community dataset using the *UNOISE3* denoising algorithm with the indicated values of stringency-controlling parameter α.

MOLECULAR ECOLOGY
RESOURCES

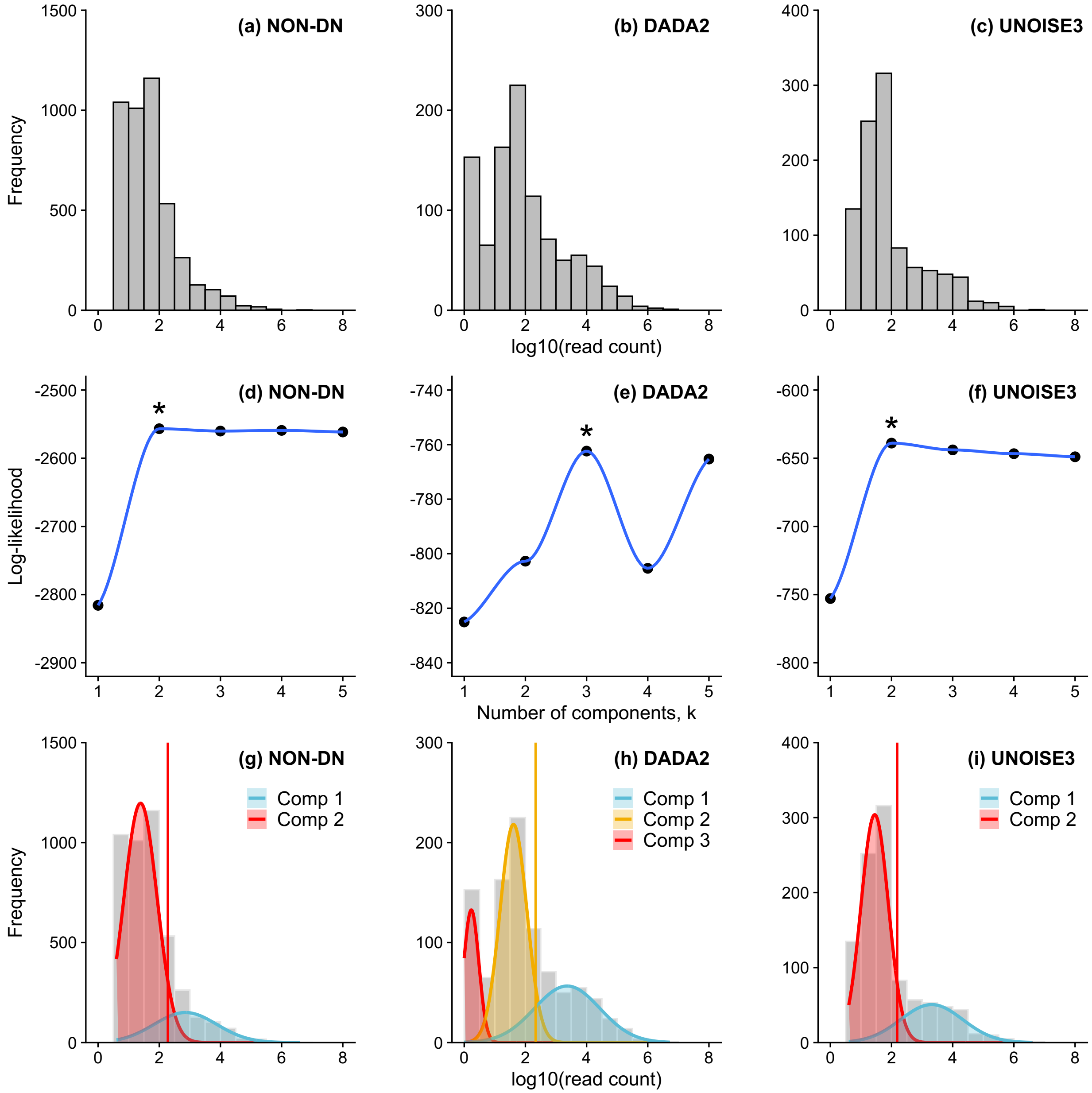

FIGURE S2 *gmmDenoise* analysis of amplicon sequence variants (ASVs) from the single-species dataset 2 processed with (a, d, g) no denoising, (b, e, h) *DADA2* denoising, and (c, f, i) *UNOISE3* denoising. For each denoising option, visual inspection of read size distribution (a, b, c) and cross-validation result (d, e, f) were used to select the number of mixture components, *k* (marked with an asterisk), with which a Gaussian mixture model was fitted (g, h, i) to infer a statistically validated cutoff threshold for error filtering, i.e., the upper one-sided 95% confidence limit of the second uppermost component (Comp 2), indicated by a vertical line.

MOLECULAR ECOLOGY RESOURCES

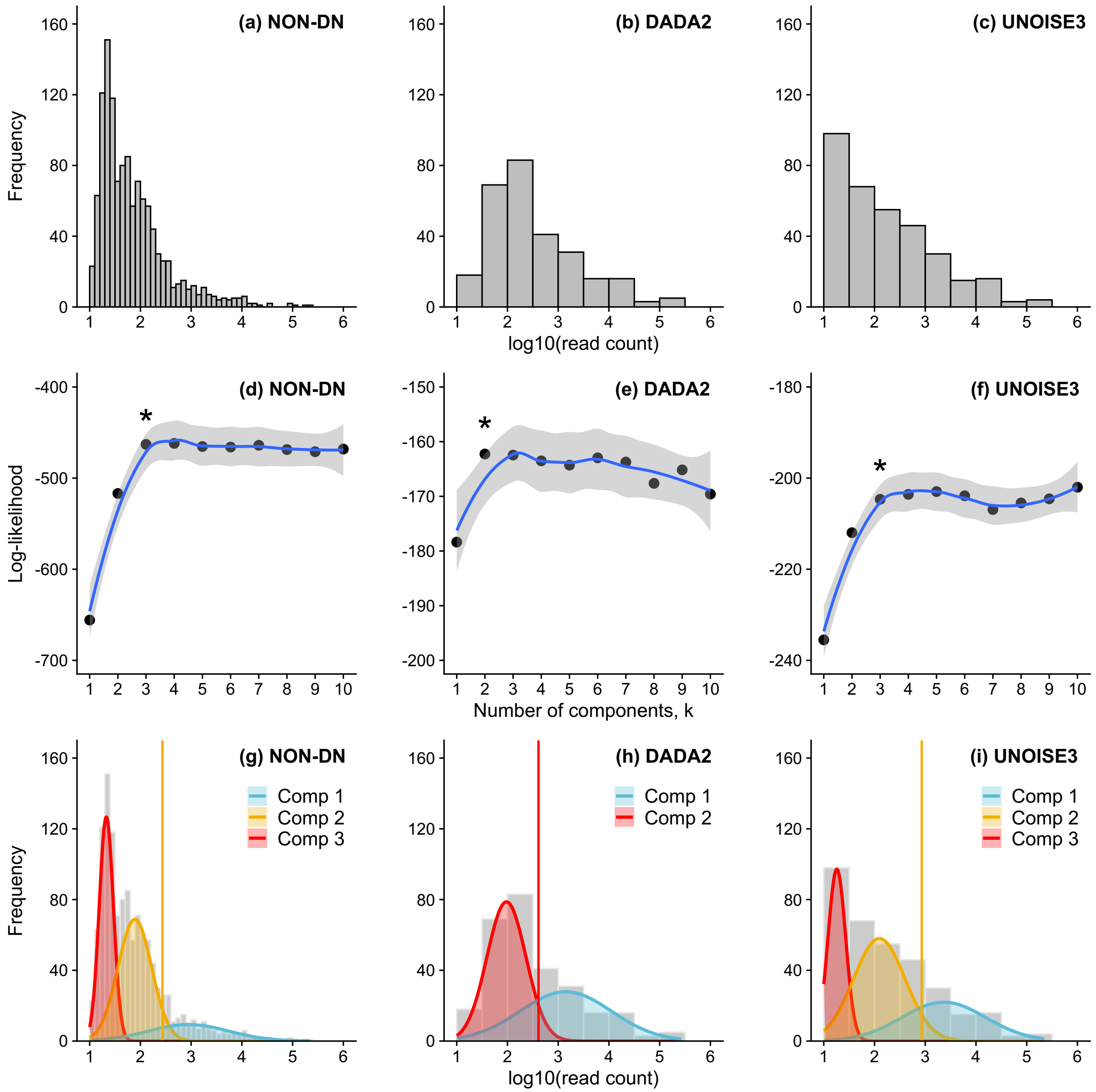

FIGURE S3 *gmmDenoise* analysis of fish amplicon sequence variants (ASVs) from the stream fish community dataset processed with (a, d, g) no denoising, (b, e, h) *DADA2* denoising, and (c, f, i) *UNOISE3* denoising. For each denoising option, visual inspection of read size distribution (a, b, c) and cross-validation result (d, e, f) were used to select the number of mixture components, *k* (marked with an asterisk), with which a Gaussian mixture model was fitted (g, h, i) to infer a statistically validated cutoff threshold for error filtering, i.e., the upper one-sided 95% confidence limit of the second uppermost component (Comp 2), indicated by a vertical line.

MOLECULAR ECOLOGY RESOURCES

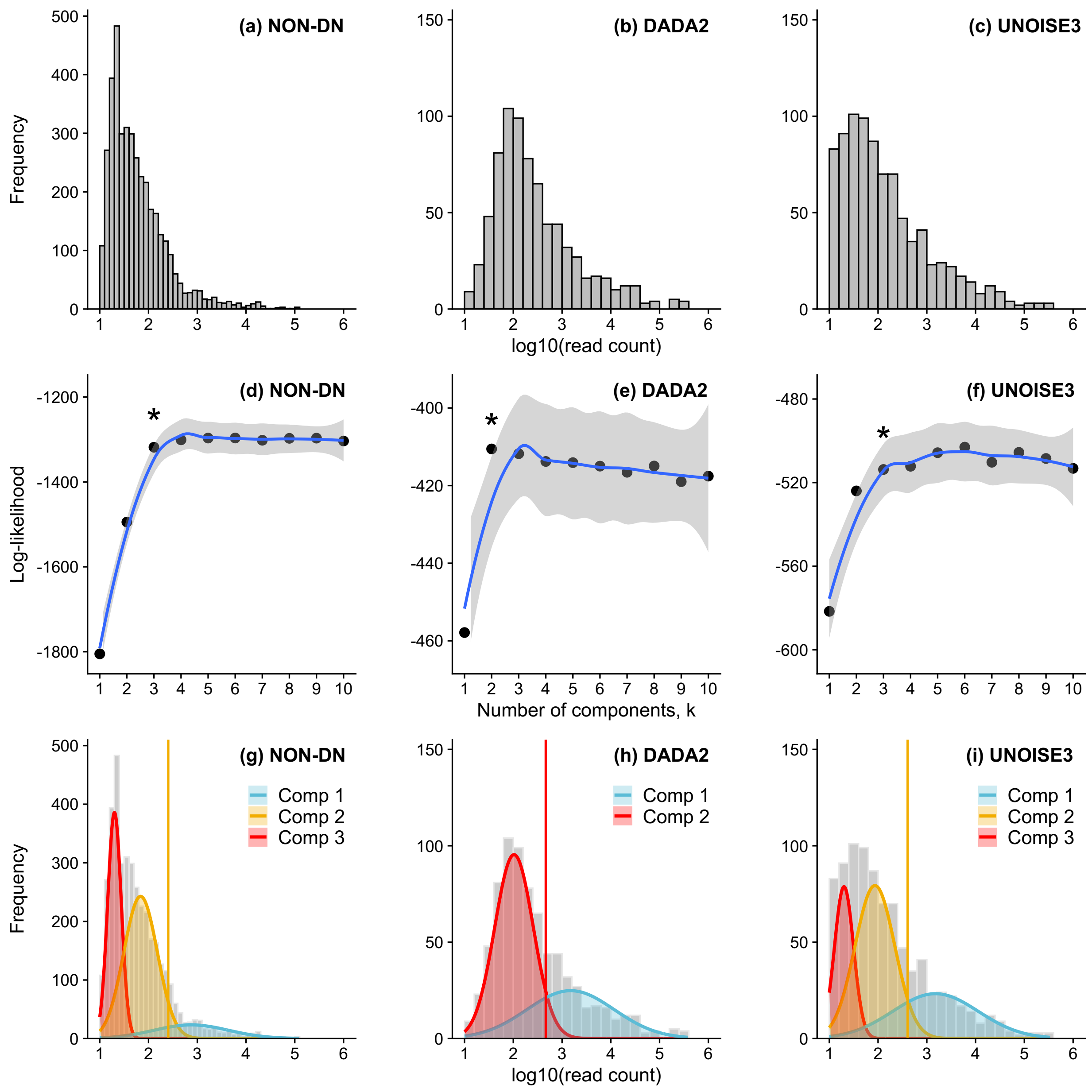

FIGURE S4 *gmmDenoise* analysis of fish amplicon sequence variants (ASVs) from the estuarine fish community dataset processed with (a, d, g) no denoising, (b, e, h) *DADA2* denoising, and (c, f, i) *UNOISE3* denoising. For each denoising option, visual inspection of read size distribution (a, b, c) and cross-validation result (d, e, f) were used to select the number of mixture components, *k* (marked with an asterisk), with which a Gaussian mixture model was fitted (g, h, i) to infer a statistically validated cutoff threshold for error filtering, i.e., the upper one-sided 95% confidence limit of the second uppermost component (Comp 2), indicated by a vertical line.
